# Expanding the spectrum of *NUS1*-related progressive myoclonic epilepsy: a novel variant and exploratory use of metformin

**DOI:** 10.3389/fgene.2025.1665623

**Published:** 2025-12-18

**Authors:** Cristina Sau, Sergi López-Rodríguez, Mercè Falip, Anna Esteve-Garcia, Jacint Sala-Padró, Cinthia Aguilera, Alba Navarro-Romero, Amaia Lasa-Aranzasti, Laura Rodríguez-Bel, Guillermo Hernández-Pérez

**Affiliations:** 1 Department of Clinical Genetics, Bellvitge University Hospital, Institut d’Investigació Biomèdica de Bellvitge (IDIBELL), L’Hospitalet de Llobregat, Barcelona, Spain; 2 Department of Psychiatry, Bellvitge University Hospital, Institut d’Investigació Biomèdica de Bellvitge (IDIBELL), L’Hospitalet de Llobregat, Barcelona, Spain; 3 Department of Neurology, Bellvitge University Hospital, Institut d’Investigació Biomèdica de Bellvitge (IDIBELL), L’Hospitalet de Llobregat, Barcelona, Spain; 4 Genetics Laboratory, Metropolitan South Clinical Laboratory, Bellvitge University Hospital, Institut d’Investigació Biomèdica de Bellvitge (IDIBELL), L'Hospitalet de Llobregat, Barcelona, Spain; 5 Neurology Area, Health in Code, Valencia, Spain; 6 Department of Clinical and Molecular Genetics, Hospital Universitari Vall d’Hebron, Vall d’Hebron Institut de Recerca (VHIR), Medicine Genetics Group, Barcelona, Spain; 7 Nuclear Medicine Department, ICS-IDI, Bellvitge University Hospital, L’Hospitalet de Llobregat, Barcelona, Spain

**Keywords:** *NUS1*, progressive myoclonic epilepsy, whole exome sequencing, *NUS1*-related disorders, psychiatric manifestations, metformin

## Abstract

**Introduction:**

Progressive myoclonic epilepsies (PME) are rare genetic disorders typically presenting with myoclonus, seizures, and cognitive decline. While several genes are associated with PME, the *NUS1* gene has recently emerged as a potential cause. We report the case of a 41-year-old woman who presented with tics, myoclonus, and language difficulties followed by gait instability, tremor, absence seizures, and psychotic symptoms including persistent hallucinations and delusional misidentification.

**Methods:**

Neurology and psychiatry specialists reviewed clinical data. Brain MRI, scalp video-EEG monitoring, and [18F]-FDG-PET/MRI were performed following standardized protocols. Whole exome sequencing (WES) guided by human phenotype ontology (HPO) terms was performed, and variants were interpreted according to American College of Medical Genetics and Genomics (ACMG) guidelines. Additionally, we conducted a review of previously reported cases of *NUS1* pathogenic/likely pathogenic variants associated with PME to better characterize the clinical and paraclinical features and to explore potential management strategies.

**Results:**

A novel heterozygous frameshift likely pathogenic variant in the *NUS1* gene, c.248del, p. (His83Profs*22), was identified in the patient. This finding led to the introduction of a targeted therapeutic strategy, including the initiation of metformin and a thorough revision of the patient’s existing psychiatric treatment. The patient showed an improvement in her psychiatric manifestations. However, neurological examination revealed either stable or slightly worsened signs, and she did not achieve seizure freedom.

**Discussion:**

This is the first review of *NUS1* from a PME perspective and the first report describing the exploratory use of metformin as a potential therapeutic intervention. In our case, metformin was introduced simultaneously with a change in antipsychotic treatment, so its specific clinical impact cannot be determined. Additional studies are needed to improve understanding of the benefits of using metformin and other therapeutic strategies in *NUS1*-related disorders. Further studies are essential to clarify the full phenotypic spectrum associated with *NUS1* variants and to improve our understanding of how specific variant types and locations contribute to clinical presentation.

## 1 Introduction

Progressive myoclonic epilepsies (PME) are a group of rare genetic and metabolic disorders that typically manifest in childhood or adolescence, characterized by action myoclonus, bilateral tonic-clonic seizures, variable ataxia, and progressive cognitive decline (Berkovic et al., 1986; Canafoglia et al., 2021). Despite their heterogeneity, PMEs can be broadly categorized into two main groups. The first group is marked by preserved cognition, with predominant bilateral tonic-clonic seizures, drug-resistant and disabling myoclonus, and ataxia, with Unverricht-Lundborg Disease (ULD) being the most common cause. The second group presents significant cognitive and psychiatric deterioration, with Lafora disease as the most representative example (Courage et al., 2021).

PMEs are among the most genetically well-characterized epilepsies, with nearly 50 causal genes identified to date (Courage et al., 2021; Cameron et al., 2023). Genetic testing can reveal the underlying genetic cause in approximately 70%–80% of PME cases (Cameron et al., 2023; Fran et al., 2014). The most frequently implicated conditions include ULD, Lafora disease, neuronal ceroid lipofuscinoses (NCL), and myoclonic epilepsy with ragged-red fibers (MERRF) (Cameron et al., 2023). However, recent studies have identified novel pathogenic variants in genes not previously associated with PME but linked to other neurological disorders involving seizures and ataxia (Courage et al., 2021; Zimmern and Minassian, 2024; Van Der Veen et al., 2024). Among these, pathogenic variants in *NUS1* have emerged as a potential cause of PME (Zimmern and Minassian, 2024).

The *NUS1* gene, located on chromosome 6, encodes the Nogo-B receptor (NgBR), which stabilizes the dehydrodolichyl diphosphate synthase complex in the endoplasmic reticulum, promoting its enzymatic activity (cis-PTase). Biallelic pathogenic variants in *NUS1* were first described in 2014 in two siblings with a congenital glycosylation defect (OMIM # 617082) (Park et al., 2014). Fibroblasts from these patients exhibited N-glycosylation defects and lysosomal cholesterol accumulation, resembling the pathogenic mechanisms of Niemann-Pick disease caused by pathogenic variants in *NPC1* and *NPC2* (Yu et al., 2021). Since then, an increasing number of fully penetrant autosomal dominant *NUS1* variants have been identified worldwide (Riboldi et al., 2022). Heterozygous *NUS1* pathogenic variants have been linked to autosomal dominant intellectual disability type 55 with seizures (OMIM # 617831) (Park et al., 2014; Li et al., 2024), complex dystonia, cerebellar ataxia, tremors, epileptic and developmental encephalopathies, Parkinson’s disease (PD), and PME (Courage et al., 2021; Zimmern and Minassian, 2024; Yu et al., 2021; Li et al., 2024), underscoring the gene’s critical role in the neuronal and neuromuscular systems. It has also been reported in association with a newly described syndrome called progressive myoclonus ataxia (PMA), which typically manifests in childhood with progressive ataxia and myoclonus, without significant cognitive decline. Seizures in this syndrome are absent, infrequent, or responsive to treatment, distinguishing it from progressive myoclonus epilepsy (PME), in which seizures are a hallmark symptom of the disease (Van Der Veen et al., 2024; Van Der Veen et al., 2018).

Despite the growing number of reported patients with diverse phenotypes associated with *NUS1* pathogenic variants, the underlying disease mechanisms remain poorly understood (Riboldi et al., 2022; Li et al., 2024; Van Der Veen et al., 2018; Hu et al., 2023). Furthermore, no targeted or personalized treatment strategies have been established for *NUS1*-related PMEs, highlighting a critical gap in clinical management.

The aim of this study is to describe a 41-year-old woman carrying a novel heterozygous *NUS1* likely pathogenic variant who presented with PME, as she exhibited myoclonus, tremors, ataxia, and psychiatric symptoms. Following confirmation of the diagnosis through molecular analysis, metformin was introduced as part of the treatment strategy, marking the first reported use of this agent in a *NUS1*-related disorder. Through comprehensive clinical and paraclinical evaluation, including a 1-year follow-up to assess treatment response, this case expands the phenotypic spectrum associated with *NUS1*-related PME. Additionally, we performed a systematic review of previously reported cases with *NUS1* variants and PME, yielding insights into potential management strategies and contributing to the growing understanding of this emerging genetic etiology.

## 2 Materials and methods

### 2.1 Clinical evaluation

The proband was under long-term care by the Epilepsy and Psychiatry Units at Bellvitge University Hospital. She underwent a comprehensive clinical assessment conducted by MF-GH (neurologists) and SL (psychiatrist). Neuroimaging studies were reviewed by LR (nuclear medicine specialist). Given the complexity of her clinical presentation and the absence of a definitive diagnosis, she was referred to the Clinical Genetics Unit for further evaluation and to guide genetic testing.

### 2.2 Paraclinical studies

MRI was performed using a 3T scanner following an epilepsy protocol. Video-EEG monitoring with scalp EEG was recorded using a 10–20 system. Interictal EEG was analyzed for generalized and focal epileptiform discharges, background slowing, and photo paroxysmal responses. [18F]-Fluorodeoxyglucose positron emission tomography/magnetic resonance (FDG-PET/MRI) was conducted following a standardized protocol. PET images were analyzed for focal or generalized metabolic alterations including cortical, subcortical, and cerebellar hypometabolism or hypermetabolism.

### 2.3 Whole exome sequencing and analysis

After a multidisciplinary team discussion, the decision was made to perform whole exome sequencing (WES) guided by Human Phenotype Ontology (HPO) annotation. The documented HPO terms for the patient included Myoclonus (HP:0001336), Myoclonic spasms (HP:0003739), Tics (HP:0100033), Tremor (HP:0001337), Abnormality of movement (HP:0100022), Involuntary movements (HP:0004305), Seizure (HP:0001250), Abnormal hippocampus morphology (HP:0025100), Intellectual disability (HP:0001249), Abnormal emotional state (HP:0100851), Atypical behavior (HP:0000708), Psychotic episodes (HP:0000725), Extrapyramidal muscular rigidity (HP:0007076), and Psychosis (HP:0000709). Additionally, given that *CSTB* was determined to be the primary candidate gene, testing for this gene was specifically requested due to its clinical significance.

WES was conducted with an xGen Exome Panel v2.0 kit (Integrated DNA Technologies, Inc, Iowa, USA). The resulting genomic library was sequenced on a NovaSeq 6000 sequencing system (Illumina, San Diego, United States). Subsequent bioinformatic analysis was conducted using the Data Genomics Exome pipeline (version v22.4.0) developed by Health in Code in Valencia, Spain. For Copy Number Variation (CNV) analysis, the Varseq software by Golden Helix (Inc, in Montana, United States) was used. WES data was filtered by a virtual panel compromising 2878 genes (Supplementary Material) associated with the HPO terms identified by the clinicians.

The classification and interpretation of genetic variants was guided by clinical, genetic, and population-based criteria in accordance with the guidelines established by the American College of Medical Genetics (ACMG) (Richards et al., 2015). The final report included only variants with established clinical relevance to the patient’s phenotype.

### 2.4 Treatment with metformin

The patient was evaluated at baseline (before the initiation of treatment), 6 months, and 1 year after treatment initiation to assess the therapeutic response to metformin and other prescribed medications (Table 1). Clinical assessments focused on the severity of myoclonus, performance on tandem gait and Romberg test, tremor intensity, and neuropsychiatric symptoms, including hallucinations, irritability, and rigidity. In addition, EEG and FDG-PET were conducted at each time point to monitor changes in cortical activity and brain metabolism.

**TABLE 1:** Clinical and paraclinical characteristics before and after treatment with metformin.

Clinical and paraclinical characteristics	Before treatment	6 months after treatment	12 months after treatment
Myoclonia (n°/min) - Head - Left hand - Right hand - Left leg - Right leg	10 63 32 58 36	9 62 64 70 39	10 27 58 12 29
Tandem and romberg	Impossible	Stable in romberg position	Stable in romberg Two steps on the tandem
Tremor	Distal and mild	No tremor	Mild (distal and action)
Hallucinations (with clinical impact)	Present	Absent	Absent
Irritability/rigidity	Moderate	Mild	Mild
FDG-PET	Slight global cortical hypometabolism, more significant in the lateral occipital cortex and primary visual. Relative increase in metabolic activity in the basal ganglia	Without changes	Global diffuse cortical hypometabolism, affecting prefrontal cortex (greater involvement than in previous studies), precuneus, lateral occipital, and primary visual cortex. Relative increase in metabolic activity in the basal ganglia
EEG (standard 30 min)	Biposterior alpha rhythm 9 Hz Irregular continuous generalized theta slowing moderate-low amplitude. Irregular intermittent slowing in the delta range, ocational bifrontal SW.	Biposterior alpha rhythm 9 Hz Irregular continuous generalized theta slowing moderate-low amplitude. Irregular intermittent slowing in the delta range, absence of interictal epileptiform activity	Biposterior alpha rhythm 8 Hz Irregular continuous generalized theta slowing moderate-low amplitude. Irregular intermittent slowing in the delta range, absence of interictal epileptiform activity
Treatment	Valproate 600 mgr/day, topiramate 125 mgr/day, quetiapine 200 mgr/day, escitalopram 20 mgr/day, lurasidone 74 mgr/day	Valproate 600 mgr/day, topiramate 125 mgr/day, quetiapine 150 mgr/day, escitalopram 20 mgr/day, lurasidone 102.5 mgr/day, metformin 850 mgr/day	Valproate 900 mgr/day, topiramate 125 mgr/day, quetiapine 50 mgr/day, escitalopram 20 mgr/day, lurasidone 74 mgr/day, metformin 850 mgr/day

Abbreviations: mgr: miligrams, min: minutes, n°: number, S: spike, W: wave.

The study received ethical approval (number PR130/25) from the Ethical Committee of University Hospital of Bellvitge and adhered to the principles outlined in the Declaration of Helsinki (World Medical Association Declaration of Helsinki, 2013). Informed consent was obtained from the patient for genetic analysis, treatment with metformin, and publication of this manuscript.

### 2.5 Review of previously published *NUS1* cases with PME

A systematic review was conducted by GH and MF using PubMed and Web of Science to identify all published articles containing the terms “NUS1” and “epilepsy.” The search was further limited to articles published in English, Spanish, or French.

From 2014 to the preparation of this manuscript, 28 publications met the initial inclusion criteria. After full-text review, 12 articles were excluded for focusing on pharmacological mechanisms without describing clinical phenotypes (1), being based on non-human models (1), presenting clinical phenotypes unrelated to epilepsy (7), or describing patients with large deletions which included other morbid genes apart from *NUS1* (3).

Ultimately, 16 articles were included in the final analysis (Table 2). These were reviewed to characterize the clinical phenotypes of 49 patients with epilepsy associated with *NUS1* gene variants. The review also examined complementary diagnostic findings, including EEG, brain MRI, and FDG-PET results, the types of genetic variants reported, the presence of additional clinical features beyond epilepsy, and the therapeutic strategies implemented.

**TABLE 2:** Clinical and genetic characteristics of patients in published studies with *NUS1* variants and PME.

Pat	References	Gender	Genetic variant	Zygosity	Variant classification	Segregation analysis	Age at epilepsy onset	EEG	Seizure type/s	Other symptoms	Cognitive impairment	Brain MRI	Treatment	Response to treatment
1	Pat 1 (Den et al., 2019)	F	c.691 + 1G>A	Het	P	*De novo*	9 m	Fast GSW (3 Hz) and slow GSW (2.5 Hz)	BTC	Tremor, ataxia, scoliosis	Mild/Moderarate	Normal	VPA, LEV	Partial
2	Pat 2 (Den et al., 2019)	M	c.691 + 1G>A	Het	P	*De novo*	8 years	Normal	Myoclonic, BTC	Ataxia, scoliosis	Moderate	Normal	Baclofen	Partial
3	Pat 1 (Gunzler and DeBrosse, 2021)	F	c.104G>A; p. (Trp35*)	Het	P	Unknown	10 years	Slow GSW (2-2.5 Hz)	Atypical absences, myclonic, BTC	Tremor, ataxia, dystonia, disartria	Mild/Moderare	Normal	N/A	N/D
4	Pat 1 (Courage et al., 2021)	F	c.310del; p. (Val104*)	Het	LP	*De novo*	4 years	N/A	Absences Myoclonic	N/A	Moderate	N/A	N/A	N/D
5	Pat 1 (Zhang et al., 2021)	M	c.51_54del; p. (Leu18Thrfs*31)	Het	P	*De novo*	3 years	Slow GSW (2-2.5 Hz)	Absences, myoclonic, BTC	Tremor, ataxia	Moderate	Normal	OXC	Partial
6	Pat 1 (Riboldi et al., 2022)	F	c.868C>T; p. (Arg290Cys)	Het	LP	*De novo*	13 years	Difuse fast activity	Focal and generalized	Dysartria, myoclonus ataxia	Mild/moderate	Cerebellar atrophy	TPM, ZNS	Good seizure control
7	Pat 1 (Hu et al., 2023)	F	c.791 + 6T>G	Het	P	*De novo*	6 years	Fast GSW (3-3.5 Hz)	Myoclonic	Tremor	Mild	Normal	LEV, VPA, PER, ketogenic diet	Ketogenic diet effective
8	Pac 1 (Li et al., 2024)	F	c.750del; p. (Leu251*)	Het	LP	*De novo*	8 years	Bitemporal and posteriors S and SW	Myoclonic	Tremor	Moderate	Normal	LEV, VPA	Partial
9	Pat 17 (Hamdan et al., 2017)	M	c.743del; p. (Asp248Alafs*4)	Het	Not specified	*De novo*	12 m	Bifrontal S	Myoclonic, BTC	Ataxia, GDD	Severe	Normal	LEV	Good seizure control
10	Pat 18 (Hamdan et al., 2017)	M	c.128_141dup; p. (Val48Profs*7)	Het	Not specified	*De novo*	10 m	Bifrontal and GSW	Myoclonic, absensce, BTC	Tremor, GDD, ASD, ADHD	Moderate	Normal	VPA, LTG, LEV; ETH, CZP, CBZ, stiri, CLB	Seizures control with VPA and CLB
11	Pat 19 (Hamdan et al., 2017)	F	Exon 2 deletion (1.3 kb)	Het	Not specified	*De novo*	2.5 years	GSW and posterior SW	Myoclonic, absence	Tremor, dysartria, motor delay	Moderate	Normal	VPA, LEV, CLB, FEL, LTG, CZP	Seizures controlled with VPA, LTH and CZP
12	Pat 6 (Fraiman et al., 2021)	M	c.692-1G>A	Het	P	*De novo*	13	Bitemporal S	Myoclonic, BTC	Tremor, dysartria, jerky hand movements, DD, saccadic intrusions and oscillations	Moderate	Thickening of the corpus callosum	LEV	Partial control
13	Pat 2 (Araki et al., 2020)	F	c.22_23insA; p. (Val8fs*126)	Het	P	Inherited	41 years	Fast GSW (3 Hz)	Myoclonic	Tremor, ataxia, dyslipidemia	No	Normal	VPA, CZP	Good seizure control
14	Pat 3 (Araki et al., 2020)	M	c.22_23insA; p. (Val8fs*126)	Het	P	Inherited	5 years	Fast GSW (3 Hz)	Absence, BTC	Tremor, ataxia, dyslipidemia, scanning speech	Mild	Cerebelar atrophy	VPA, TPM, CZP	Good seizure control
15	Pat 4 (Araki et al., 2020)	F	c.22_23insA; p. (Val8fs*126)	Het	P	Inherited	7 years	Fast GSW (3 Hz)	Absence	Tremor, ataxia	Mild	Normal	VPA	Good seizure control
16	Pat 5 (Araki et al., 2020)	F	c.22_23insA; p. (Val8fs*126)	Het	P	Inherited	5 years	Fast GSW (3 Hz)	Absence	Tremor, ataxia, scanning speech	Moderate	Cerebelar atrophy	VPA	Good seizure control
17	Pat 1 (Ji et al., 2023)	M	c.302T; p. (Met101Lys)	Het	LP	*De novo*	6 years	GSW	Myoclonic, BTC	Ataxia	Mild	Normal	VPA, CZP	Good seizure control
18	Pat 1 (Park et al., 2014)	M	c.869G>A; p. (Arg290His)	Hom	Not specified	Inherited	11 m	N/A	BTC	IGR, hypotonia, congenital scoliosis, DD, cognitive regression. Died 1st year	Not specified	N/A	Unknown	Unknown
19	Pat 2 (Park et al., 2014)	M	c.869G>A; p. (Arg290His)	Hom	Not specified	Inherited	7 m	N/A	N/A	Hypotonia, motor delay, scoliosis, hypertrichosis, microcephaly, spasticity, visual and hearing impairment	Not specified	Cortical atrophy	Unknown	Unknown
20	Pat 1 (Yu et al., 2021)	F	c.734G>T; p. (Gly245Val)	Het	P	*De novo*	2 years	N/A	Absence	Tremor, ataxia, ocular flutter, speech delay	Borderline	Normal	Unknown	Unknown
21	Pat 2 (Yu et al., 2021)	F	c.752T>G; p. (Leu251*)	Het	P	*De novo*	4 years	Left temporal S	Absence, BTC	Tremor, ataxia, speech and developmental delay	Mild	Normal	Unknown	Unknown
22	Pat 3 (Yu et al., 2021)	M	c.415 + 1G>A	Het	LP	*De novo*	2 years	GSW	Myoclonic, absence	Tremor, DD, speech delay, dysarthria	Not specified	Subcortical parietal gliosis	Unknown	Partial
24	Pat 6 (Williams et al., 2024)	n/a	c.472G>T; p. (Glu158*)	Het	P	*De novo*	Unknown	Slow GSW (2-2.5 Hz)	N/A	Termor, ataxia, speech delay, GDD, mild bilateral SNHL	Moderate	Subtle increased signal in dentate nuclei	Unknown	Unknown
25	Pat 1 (Shen et al., 2024)	F	c.868C>T; p. (Arg290Cys)	Het	LP	*De novo*	5 years	Slow GSW (2-2.5 Hz)	Absence, tonic	DD	Moderate	Normal	VPA, LTG	Good seizure control
26	Pat 2 (Shen et al., 2024)	M	c.792–2A>G	Het	LP	*De novo*	10 m	Slow and Fast GSW (2–3 Hz), GPSW, temporal S	Tonic, focal myoclonic	Unknown	Mild	Normal	VPA	Seizure-free
27	Pat 1 (Brooker et al., 2025)	M	c.99dup; p. (Asn34Glufs*100)	Het	P	Unknown	1,5 years	Mild to moderate diffuse slowing, eyelid myoclonia without EEG change	Myoclonic, myoclonic-astatic, GTCS	GDD, myoclonus ataxia plus parkinsonism, spasticity, dysarthria, psychiatric manifestations	Severe	N/A	N/A	N/A
28	Pat 2 (Brooker et al., 2025)	F	c.218_242del; p. (Arg73Hisfs*24)	Het	P	*De novo*	2 years	Fast GSW (3–4 Hz)	Eyelid myoclonic, myoclonic, GTCS	Dystonia, spasticity, dysarthria, regression	Mild	N/A	TPM, CBD, CLB, CLN, LEV, LTG, PHN, VPA, VGB, ZNS	Partial seizure control
29	Pat 3 (Brooker et al., 2025)	M	c.238_263del; p. (Ala80Argfs*45)	Het	P	*De novo*	1 year 7 m	OIRDA, slow GSW (2–2.5 Hz) until 6 years. Fast GSW (3–3.5 Hz) at 13 y	Eyelid myoclonic, myoclonic astatic, absence, NCSE	Mild ataxia, intention tremor, GDD, hypotonia, scoliosis, psychiatric manifestations	Mild	Unilateral hippocampus hypoplasia	ETX	Seizure free
30	Pat 4 (Brooker et al., 2025)	F	c.26G>A; p. (Trp9*)	Het	P	Inherited	2 years	Background slowing, slow GSW (2–3 Hz)	Febrile seizures, tonic seizures, GTCS	Dysarthria, microcephaly, ataxia, dystonia, parkinsonism, GDD	Mild/moderate	N/A	TPM, CLB, LMT	Seizure free
31	Pat 5 (Brooker et al., 2025)	F	c.118_119insGCTGCCGCGCCGCC; p. (Ser45Alafs*10)	Het	P	*De novo*	4.5 years	Left hemisphere slowing, GSW	Focal, FBTCS	Spasticity, asymmetric muscle weakness, GDD	Severe	Corpus callosum abnormality	VPA, LEV	N/A
32	Pat 6 (Brooker et al., 2025)	F	c.671A>G; p. (Asp224Gly)	Het	LP	*De novo*	4 years 7 m	No inter-ictal activity	Absence	Axial hypotonia, dysarthria, hypothyroidism, atopic dermatitis, speech delay	Mild	N/A	LMT, VPA	Seizure free
33	Pat 11 (Brooker et al., 2025)	F	c.767G>A; p. (Trp256*)	Het	LP	*De novo*	2 years	Slow GSW (2.5–3 Hz), hyperventilation reactivity	Absence seizures after hyperventilation	Speech delay, GDD, myoclonus, ataxia, dysarthria	Mild	N/A	VPA	No response
34	Pat 12 (Brooker et al., 2025)	M	c.824del; p. (Glu275Glyfs*29)	Het	LP	*De novo*	3 m	GSW	Febrile sizure, GTCS	Severe speech delay, mild motor delay, mild ataxia, intention tremor, behavioral problems	Mild/moderate	N/A	VPA	Seizure free
35	Pat 13 (Brooker et al., 2025)	F	c.814del; p. (Ile272Serfs*32)	Het	LP	*De novo*	6 m	Background slowing, GS	Tonic-clonic	Mild GDD, mild ataxia and intention tremor	Mild	N/A	LEV	Seizure-free
36	Pat 15 (Brooker et al., 2025)	M	c.220_244del; p. (Gly74Thrfs*23)	Het	P	*De novo*	1 year	Background slowing, slow GSW (2–3 Hz)	Absence, eyelid myoclonic, NCSE	GDD, mild ataxia, eating disorder	Mild	N/A	VPA, sultiame	Partial response
37	Pat 16 (Brooker et al., 2025)	M	c.415 + 1G>A	Het	P	Unknown	Neonatal	N/A	Generalized onset seizure (no more detailes available)	Myoclonus, ataxia, dysarthria, mild hearing loss, difficulty regulating emotions	Mild	Cerebellar atrophy	VPA	Partial response
38	Pat 17 (Brooker et al., 2025)	F	Whole gene deletion (6q22.1; Hg19 chr6:117950297_118016721). Deletion of exons 1-3 and intergenic region	Het	P	*De novo*	2 years	Background slowing, frontal slowing	Absence, eyelid myoclonia	Myoclonus, ataxia plus parkinsonism, pyramidal tract signs, anxiety	Mild	N/A	VPA, sultiame	Partial response
39	Pat 20 (Brooker et al., 2025)	F	c.416–2A>C	Het	LP	*De novo*	23 years	Normal	Tonic-clonic	Mild DD, mild speech delay, myoclonus ataxia, dysarthria, anxiety	Mild	Diffuses cortial atrophy	VPA, ZNS	Seizure-free
40	Pat 24 (Brooker et al., 2025)	M	c.472G>T; p. (Glu158*)	Het	P	*De novo*	10 years	Fronto-parietal S	Focal	GDD, myoclonus ataxia, mild dysarthria, mild sensorineural hearing loss	Moderate	N/A	VPA	Seizure free
41	Pat 28 (Brooker et al., 2025)	F	c.416-1453_541 + 180del; p. (Gly139_Gln180del)	Het	P	*De novo*	20 m	Abnormal background with age (>7a), GSW	Eyelid myoclonia, absence seizures, tonic-clonic	Not specified	Not specified short	N/A	LEV, CBD, VPA	Poor response
42	Pat 29 (Brooker et al., 2025)	M	c.128_141del; p. (Ala43Glyfs*86)	Het	P	*De novo*	4 years	Occipital S, GSW	Febrile seizure, tonic-clonic	Mild DD, dysarthria, attention span	Mild	N/A	VPA	Partial response
43	Pat 30 (Brooker et al., 2025)	M	c.52_53del; p. (Leu18Alafs*115)	Het	P	*De novo*	4 years	Left fronto-parietal S	Tonic-clonic	Severe speech delay, mild DD, hypotonia, severe dysarthria, speech apraxia	Mild	Corpus callosum abnormality	LEV, LTG, VPA	N/A
44	Pat 31 (Brooker et al., 2025)	M	c.132_154del; p. (Ser45Argfs*81)	Het	P	*De novo*	2 years	GPS	Tonic-clonic	GDD, myoclonus, dystonia, tics, bilateral horizontal nystagmus	Mild	N/A	LEV	Seizure-free
45	Pat 33 (Brooker et al., 2025)	F	c.128_141dup; p. (Val48Profs*7)	Het	P	*De novo*	3 years	GSW	Febrile seizure	Myoclonus	Not specified	N/A	N/A	N/A
46	Pat 34 (Brooker et al., 2025)	M	c.293C>G; p. (Pro98Arg)	Het	LP	*De novo*	2.5 years	GSW	Focal, FBTC	GDD	Moderate	N/A	CBZ, LCM	Partial response
47	Pat 37 (Brooker et al., 2025)	M	c.868C>T; p. (Arg290Cys)	Het	LP	Unknown	2.5 years	Background slowing, GSW	Absence, atonic, myoclonic, GTCS	Isolated ataxia, dysarthria, severe behavioral issues	Severe	N/A	CLB, ZNS	Poor response
48	Pat 38 (Brooker et al., 2025)	F	c.622C>T; p. (Cys208Arg)	Het	LP	Unknown	29 years	N/A	N/A	GDD, myoclonus	Not specified	N/A	N/A	N/A
49	Pat 39 (Brooker et al., 2025)	F	c.35del,; p. (Leu12Argfs*38)	Het	P	*De novo*	22 years	Slow GSW (2–3 Hz)	N/A	Myoclonus, anxiety	Not specified	N/A	LEV, ZNS	Poor response
50	Actual report	F	c.248del; p. (His83Profs*22)	Het	LP	Unknown	8 years	Slow GSW (2.5 Hz)	Myoclonic, absence	Tremor, ataxia, auditory allucinations, scoliosis, dysmetria	Mild	Normal	TPM, VPA, LEV Metformin	Partial response to metformin

Abbreviations: ADHD: Attention-Deficit Hyperactivity Disorder; ASD: autism spectrum disorder; BTC: Bilateral Tonic-Clonic Seizure; CBD: cannabidiol; CBZ: carbamazepine; CLB: clobazam; CZP: clonazepam; DD: developmental delay; ETS: ethosuximide; FBTCS: Focal to Bilateral Tonic-Clonic Seizure; FLB: felbamate; GDD: global developmental delay; GPSW: Generalized Poly-Spike Wave; GSW: Generalized Spike-Wave; GTCS: Generalized Tonic-Clonic Seizure; het: Heterozygous; hom: Homozygous; IGR: intrauterine growth retardation; LCM: lacosamide; LEV: levetiracetam; LP: likely pathogenic; LTG: lamotrigine; N/A: not applicable; N/D: not determined; NCSE: non-convulsive status epilepticus; P: pathogenic; Pat: Patient; PHE: phenytoin; S: spikes; SNHL: sensorineural hearing loss; STR: stiripentol; SW: Spike-Wave; TPM: topiramate; VGB: vigabatrin; VPA: valproate; ZNS: Zonisamide. NM_138459.3.

## 3 Results

### 3.1 Clinical findings

Our patient, a 41-year-old right-handed woman, was the only child of non-consanguineous parents. She had an uneventful birth and normal neurodevelopment until the age of 8. She had no history of febrile seizures, central nervous system infections, or head trauma, nor was there a family history of epilepsy or other neurological diseases.

The patient first exhibited symptoms at age 8, presenting with tics and myoclonus, followed by language difficulties. Despite these challenges, she was able to complete the school years with support from teachers and parents. By the age of 13, her parents reported progressive difficulties in running and walking, along with tremors that made it increasingly difficult to participate in physical activities at school. Around the same time, she began experiencing absence seizures, without other associated movement disorders.

At the time of referral to our center at age 20, neurological examination showed distal myoclonus in both upper and lower limbs, sometimes triggered by movement and mini-myoclonus of the face. The patient exhibited dysmetria in all four limbs and a tremor with an intentional component. Gait assessment revealed instability with ataxia, a widened base of support, a positive Romberg sign, and an inability to walk in tandem. The patient showed no dysmorphic features or structural abnormalities of the central nervous system, other organs, or tissues. Findings from laboratory investigations were unremarkable apart from dyslipemia that started in the early twenties. An initial EEG revealed a loss of the dominant posterior rhythm, along with high-voltage generalized spike-wave or polyspike discharges at 2–2.5 Hz. Intermittent photic stimulation did not induce changes in the EEG.

At the age of 27, she developed psychotic symptoms, initially presenting with a delusional misidentification syndrome and persistent auditory hallucinations involving dialoguing voices. There was no family history of psychiatric disorders and no previous personal psychiatric comorbidity. Since then, her clinical trajectory has been marked by fluctuating stability and persistent sensory-perceptual disturbances. Despite multiple antipsychotic treatments (quetiapine, risperidone, aripiprazole, perphenazine, pimozide) and periods of relative euthymia and functional capacity, core psychotic symptoms remained resistant, occasionally escalating to extracampine auditory phenomena and visual hallucinations. These episodes were frequently accompanied by irritability, ruminative thinking and low mood, sometimes requiring hospitalization and therapeutic reassessment.

**FIGURE 1:**
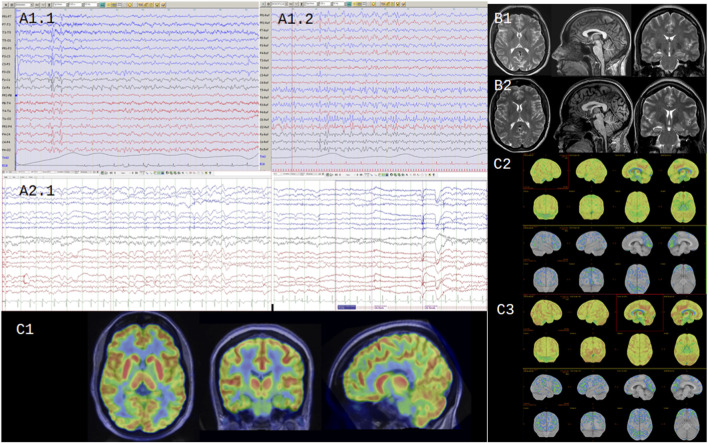
Neuroimaging and electrophysiological findings over time. **(A1.1)** EEG (2012, bipolar montage): generalized interictal epileptiform discharges with maximum electronegativity in the occipital regions. **(A1.2)** EEG (2012): intermittent photic stimulation elicits occipital driving response. **(A2.1)** EEG (2025, bipolar montage): no interictal epileptiform discharges observed throughout the recording; intermittent photic stimulation produces no changes. **(B1)** Brain MRI (2012 and 2016): no significant changes observed between the two studies. **(C1)** FDG-PET/MRI (2023): performed 30 min after intravenous injection of 4 mCi of [18F]-FDG using a Signa GE PET/MRI scanner; moderate hypermetabolism observed in the basal ganglia and thalami in axial **(A)**, coronal **(B)**, and sagittal **(C)** views. **(C2**) Quantitative PET analysis (2023): automatic 3D-SSP analysis with normalization to pontine activity identifies regions of statistically significant hypometabolism (compared to age-matched controls), shown in yellow and red on three-dimensional reconstructions. **(C3)** Quantitative PET analysis after 1 year of metformin treatment (2025): expanded areas of hypometabolism, predominantly in the frontal lobes.

Multiple brain MRIs performed over the past 20 years revealed hippocampal asymmetry without evidence of mesial sclerosis or other abnormalities. No progressive atrophic changes were detected. In 2023, an FDG-PET/MRI study showed global cortical hypometabolism and increased metabolic activity in the basal ganglia and thalamus, findings that persisted in a follow-up study 1 year later (Figure 1).

Throughout the course of her disease, various anti-seizure treatments have been tested and rotated without achieving either seizure freedom or significant reduction of the myoclonus: valproate up to 1000 mg/d in combination during the follow-up in our unit, zonisamide up to 300 mg/d in combination during 1 year, levetiracetam in combination up to 1500 mg/d for 1 year, clonazepam up to 2 mg/d for 1 year, and topiramate up to 125 mg/d at last follow-up.

### 3.2 Identification of a novel variant in the *NUS1* gene

WES analysis revealed a novel heterozygous variant in the *NUS1* gene, NM_138459.3:c.248del; p. (His83Profs*22). The variant was detected with a read depth of 30X and further confirmed by Sanger Sequencing.

**FIGURE 2:**
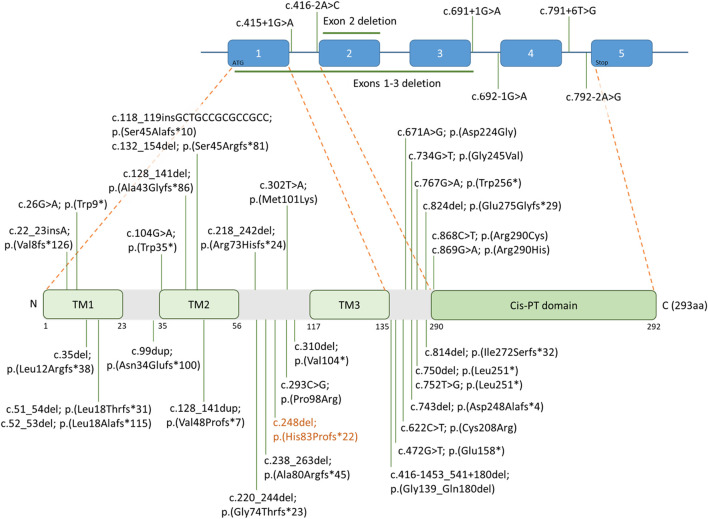
*NUS1* gene structure and location of reported variants associated with PME. This figure illustrates the exon-intron structure of the *NUS1* gene and its encoded protein, which contains three predicted transmembrane domains (TM1, TM2, TM3) and a cis-prenyltransferase (cis-PTase) domain. The novel frameshift variant identified in this study [c.248del; p. (His83Profs*22)] is shown in orange, while previously reported PME-associated variants are shown in black. Variants are positioned according to their respective locations within the gene and protein structure.

This variant consists of a single-nucleotide deletion that is expected to cause a frameshift at the protein level from amino acid 83, leading to the introduction of a premature stop codon 22 residues later. It is located in exon 1/5 within the *NUS1* gene, and the resulting mRNA is predicted to undergo degradation through nonsense-mediated mRNA decay (NMD). Figure 2 shows the position of this variant in our patient, along with all previously reported *NUS1* variants included in our review, mapped onto the gene structure and protein domains to highlight their distribution and potential functional significance.

The variant is absent from gnomAD database (v4.1.0) and has not been reported in the medical literature in association with disease. However, other truncating variants located downstream have been classified as pathogenic in clinical databases (ClinVar and HGMD) and have been identified in patients with a clinical phenotype that includes epilepsy, global developmental delay, hippocampal hypoplasia, parasomnia, myoclonus, involuntary movements, ataxia, and scoliosis, among others (Den et al., 2019).

Moreover, *NUS1* is recognized as highly intolerant to loss-of-function variants (pLI = 1) and is classified as an haploinsufficient gene (HI Score: 3, ClinGen). Given that the identified variant is located in the first exon and is predicted to trigger NMD, it is likely to result in a complete absence of functional protein from the affected allele. This supports a loss-of-function mechanism consistent with the pathogenic processes previously associated with *NUS1* variants (Yu et al., 2021; Li et al., 2024; Den et al., 2019).

Based on the currently available evidence and following the ACMG standard classification guidelines (Richards et al., 2015), this variant was classified as likely pathogenic. The main criteria used to classify this variant include PVS1-very strong (truncating variant in a gene where loss of function is a known pathogenic mechanism) and PM2-supporting (absent in gnomAD v4.1.0).

Segregation analysis revealed that the patient’s mother did not carry the c.248del; p. (His83Profs*22) variant. The patient’s father, who passed away at the age of 50 without presenting any neurological symptoms, could not be tested; thus, it remains unclear whether the variant arose *de novo* or was paternally inherited. There was no reported family history of neurological symptoms.

## 4 Discussion

### 4.1 Epilepsy

Epilepsy is a common clinical feature among individuals carrying *NUS1* variants (Brooker et al., 2025). It typically manifests during childhood, although the age of onset varies widely. Reported cases show initial seizure manifestations occurring as early as 9 months of age, with febrile and generalized tonic-clonic seizures (Den et al., 2019), and as late as 41 years (Araki et al., 2020). Most patients, however, develop epilepsy between the ages of 5 and 8 years (Courage et al., 2021; Hu et al., 2023; Ji et al., 2023), with a few presenting later, around adolescence (Riboldi et al., 2022; World Medical Association Declaration of Helsinki, 2013), or earlier in infancy (Den et al., 2019; Landais et al., 2025; Szafranski et al., 2015). Adult onset, although reported, is very unusual (Brooker et al., 2025).

Seizure types are heterogeneous, and most patients experience multiple seizure semiologies (Brooker et al., 2025). Commonly reported types include myoclonic seizures or myoclonic tremor (Courage et al., 2021; Riboldi et al., 2022; Hu et al., 2023; Ji et al., 2023; Landais et al., 2025; Szafranski et al., 2015; Haginoya et al., 2021), eyelid myoclonic (Brooker et al., 2025), generalized tonic-clonic seizures (Den et al., 2019; Araki et al., 2020; Ji et al., 2023; Haginoya et al., 2021; Zhang et al., 2021), and absence seizures, both typical and atypical (Courage et al., 2021; Araki et al., 2020; Szafranski et al., 2015), as well as tonic and clonic seizures (Araki et al., 2020; Szafranski et al., 2015). The variability in age of onset and seizure type highlights the heterogeneous nature of epilepsy in the context of *NUS1*-related neurodevelopmental disorders. Seizures are usually drug-resistant when the patient presents a PME, but they can be well-controlled or absent in other phenotypes such as PMA or while expressed as only as a movement disorder disease (Van Der Veen et al., 2024; Brooker et al., 2025).

The clinical presentation of our patient, who began experiencing absence seizures at age 13, aligns with the broad phenotypic spectrum of epilepsy previously reported in association with *NUS1* variants.

### 4.2 Movement disorders

Movement disorders are present in most individuals with *NUS1* variants and typically manifest during early childhood (Brooker et al., 2025). Myoclonus is the most frequently observed movement abnormality and often represents one of the earliest neurological symptoms potentially preceding the onset of seizures (Riboldi et al., 2022). Tremor is another prominent feature present in most patients, and may appear either before or after the development of epilepsy (Den et al., 2019; Szafranski et al., 2015; Gunzler and DeBrosse, 2021). This pattern aligns with the clinical course of our patient, who began exhibiting symptoms at the age of 8 with tics and myoclonus. She did not experience her first absence seizure until the age of 13. Notably, she also presented with facial mini-myoclonus, a feature described in other patients as well (Riboldi et al., 2022; Zhang et al., 2021).

As patients enter late childhood and adolescence, there is often a progression of neurological symptoms, including worsening myoclonus, the emergence of intentional tremor, and increasing gait difficulties characterized by ataxia (Riboldi et al., 2022; Li et al., 2024; Szafranski et al., 2015). Cerebellar ataxia is a commonly reported feature in *NUS1*-associated disease, frequently accompanied by dysarthria, whereas gait impairment is generally milder in comparison (Park et al., 2014; Brooker et al., 2025). In our case, the patient exhibited language difficulties from the onset (age 8), and by age 13 her parents noted a gradual decline in motor coordination, with increasing difficulty in running and walking, as well as tremor that interfered with physical activities at school. At present, she displays dysarthric speech and progressive gait instability that has continued to worsen over the years. She does not exhibit other neurological symptoms that are also described in *NUS1*-related disorders, such as parkinsonism or dystonia.

### 4.3 Cognitive and neurodevelopmental features

Most of the patients in whom a neuropsychological evaluation was performed showed a mild to moderate intellectual disability (Hu et al., 2023; Den et al., 2019; Ji et al., 2023; Szafranski et al., 2015; Zhang et al., 2021; Gunzler and DeBrosse, 2021; Williams et al., 2024) with only a few individuals presenting with severe impairment (Brooker et al., 2025). Although a formal intellectual assessment was not performed, our patient showed signs of mild intellectual disability, which appeared to have progressively worsened over the years. This gradual cognitive decline, beginning in childhood, aligns with previously reported cases of *NUS1*-related disorders (Courage et al., 2021; Riboldi et al., 2022; Hu et al., 2023). Previous studies have proposed that variants affecting the C-terminal domain might be associated with more severe cognitive impairment due to their potential impact on protein function (Riboldi et al., 2022). However, when considering the cases summarized in Table 2, this pattern is not consistently observed. Reported individuals with C-terminal variants show a broad spectrum of cognitive outcomes ranging from mild to severe impairment, and a similarly wide range is evident among those with N-terminal variants. These observations indicate substantial phenotypic variability irrespective of variant location; therefore, the severity of cognitive impairment cannot be reliably inferred based on the variant’s position within the gene.

In addition, some patients with *NUS1* variants exhibit broader neurodevelopmental features such as early developmental delay and hypotonia, autism spectrum disorder, and attention-deficit hyperactivity disorder, suggesting that *NUS1*-related disease may, in certain cases, fall within a wider and more complex neurodevelopmental spectrum (Yu et al., 2021; Brooker et al., 2025; Landais et al., 2025). These features are most commonly reported in individuals with deletions encompassing *NUS1* together with additional genes (Landais et al., 2025) but they have also been described in patients carrying variants affecting *NUS1* exclusively, as previously reported (Yu et al., 2021; Brooker et al., 2025; Hamdan et al., 2017; Ding et al., 2025) and as observed in our review of published cases.

Most patients carrying *NUS1* variants do not present with dysmorphic features (Li et al., 2024; Hu et al., 2023; Den et al., 2019; Szafranski et al., 2015). In the few reported cases where such traits were observed, the individuals harbored copy number variants involving *NUS1* along with other clinically significant genes (Szafranski et al., 2015). Dyslipemia from a young age is a common finding in these patients. Our patient had dyslipemia beginning in her early twenties but did not exhibit dysmorphic features or other manifestations described in some *NUS1*-related cases, such as scoliosis.

### 4.4 Psychiatric findings

Our patient presented with delusional misidentification syndrome and persistent auditory hallucinations involving dialoguing voices. Auditory hallucinations have previously been reported in the literature in a patient with a *NUS1* deletion who also exhibited symptoms of manic behavior (Landais et al., 2025), as well as in a patient carrying an intronic *NUS1* variant who presented with a broader psychiatric profile including persecutory delusions, aggression, and sleep disturbances (Fraiman et al., 2021). Additional psychiatric manifestations such as irritability or anxiety have also been described in individuals with *NUS1* variants (Brooker et al., 2025; Gunzler and DeBrosse, 2021) suggesting that certain genetic variants in *NUS1* may contribute to an increased susceptibility to psychotic symptoms.

### 4.5 Treatment with metformin

Currently, there is no established treatment protocol for managing refractory epilepsy in individuals with *NUS1*-related disorders. Seizures in these patients are frequently resistant to broad-spectrum antiepileptic drugs such as valproate, lamotrigine, levetiracetam, zonisamide, and topiramate (Hu et al., 2023; Brooker et al., 2025; Szafranski et al., 2015). This was also the case with our patient, who initially presented with absence of seizures and received various anti-seizure medications, including valproate, levetiracetam, clonazepam, and zonisamide, without achieving seizure freedom.

Published case reports reflect a similar limited and heterogeneous therapeutic response. Den et al. (2019) reported a partial response to levetiracetam and valproate, while Zhang et al. (2021) described a patient who showed significant improvement in both seizure control and language function, along with partial improvement in motor abilities, following treatment with carbamazepine. Additionally, Hu et al. (2023) highlighted the effectiveness of a ketogenic diet in improving both seizure frequency and cognitive performance. Treatment of associated movement disorders has also been reported in isolated cases: botulinum toxin has been used for severe dystonia (Gunzler and DeBrosse, 2021), deep brain stimulation has reduced tremor severity (Haginoya et al., 2021), and clonazepam, brivaracetam, levetiracetam, zonisamide, and propranolol have been reported to improve myoclonus (Brooker et al., 2025).

Metformin has been approved as an orphan drug for Lafora disease, a PME caused by a congenital glycosylation defect (Burgos et al., 2023; Papini et al., 2024). Although this condition is pathophysiologically distinct from *NUS1*-related disorders, metformin has demonstrated broader neuroprotective effects that extend beyond its use in Lafora disease. Notably, metformin modulates lysosomal cholesterol homeostasis and enhances autophagy, mechanisms that are increasingly recognized as relevant in lysosome-dependent neurodegenerative conditions such as Niemann-Pick disease (Best et al., 2025). This is particularly relevant for *NUS1*-related disease, as *NUS1* dysfunction disrupts dolichol biosynthesis and protein glycosylation, processes that can secondarily impair intracellular lipid trafficking and lysosomal function (Park et al., 2014; Yu et al., 2021). On this mechanistic basis, metformin was considered a reasonable therapy in our patient. Metformin was introduced at the same time as a switch from quetiapine to lurasidone, a previously untried antipsychotic. This change in treatment plan in our patient led to an improvement in psychiatric symptoms, with no significant changes in myoclonus or tremor, and no moderate-to-severe adverse effects (Table 1). The decision to initiate both treatments concurrently was driven by the urgent clinical need to manage severe psychotic symptoms in a patient with a complex neurogenetic disorder. In routine clinical practice, particularly in cases of this complexity, it is often necessary to implement multiple therapeutic interventions simultaneously to provide optimal patient care.

We considered using clozapine, primarily indicated for treatment-resistant schizophrenia. However, the high risk of seizure under clozapine treatment, the need for hematological monitoring, and the presence of psychotic symptoms in the context of a neurogenetic disorder without prior antipsychotic trials, led us to consider other options (Wong and Delva, 2007). Lurasidone offers a more appropriate safety profile for this clinical context, with minimal impact on seizure threshold [efficacy for both positive and negative symptoms, and additional procognitive effects mediated through 5-HT1a partial agonism and 5-HT7 antagonism (Horisawa et al., 2011; Huang et al., 2012)]. These properties were seen to be particularly beneficial given the patient’s mild intellectual disability.

This most recent period marks one of the most sustained and functionally significant improvements since the onset of the patient’s long-standing psychotic symptoms. While lurasidone has demonstrated efficacy in treating psychosis through its D2 and serotonergic antagonism, we hypothesized that metformin might contribute through mechanisms related to mitochondrial dysfunction associated with *NUS1* variants. However, we acknowledge that this remains speculative, and further studies are required to provide evidence supporting the potential therapeutic effect of the drug in this as yet orphan disease.

To our knowledge, there are no published reports on the use of hypolipidemic agents, metformin, or miglustat in patients with *NUS1*-related disorders (Best et al., 2025). However, based on the pathophysiological mechanisms implicated in these conditions, these agents may represent promising future therapeutic options. In our experience, it was well tolerated, with no adverse events observed.

### 4.6 Neurophysiological and neuroimaging features

EEG findings in *NUS1*-related disorders are commonly characterized by slowing down of background rhythm and loss of the posterior dominant rhythm and generalized slow spike-wave discharges at 2–2.5 Hz (Szafranski et al., 2015), as observed in our patient. Nevertheless, EEG patterns are variable, ranging from normal recordings (Richards et al., 2015) to focal, multifocal, or generalized abnormalities (Brooker et al., 2025). A photoparoxysmal response has been reported in only one case to date (Haginoya et al., 2021).

Neuroimaging findings also demonstrate considerable heterogeneity. Reported MRI results range from normal brain structure (Den et al., 2019; Szafranski et al., 2015) to mild cerebellar atrophy, more diffuse cortical atrophy, and abornmalities of the corpus callosum (Riboldi et al., 2022; Brooker et al., 2025). Interestingly, Haginoya et al. (2021) described a patient with glucose PET/CT showing symmetrically increased metabolism in the basal ganglia—a pattern also observed in our patient.

### 4.7 Inheritance in *NUS1*-related disorders

In most individuals with *NUS1*-related disorders, an autosomal dominant inheritance pattern due to a *de novo* variant has been reported (Li et al., 2024; Hu et al., 2023; Den et al., 2019; Haginoya et al., 2021; Williams et al., 2024). However, Araki et al. (2020) described five affected individuals from the same family carrying a *NUS1* variant who exhibited a wide spectrum of clinical phenotypes including epilepsy, tremor, ataxia, myoclonus, and intellectual disability. These findings suggest that *NUS1* variants can exhibit considerable variability in clinical expressivity, even among individuals within the same family. Furthermore, Park et al. (2014) identified a homozygous missense *NUS1* variant in two siblings with a congenital disorder of glycosylation, while their heterozygous parents and siblings remained asymptomatic, representing the only reported case to date of a possible autosomal recessive inheritance pattern. These observations highlight the clinical heterogeneity associated with *NUS1* variants, underlining the importance of thorough genetic counseling for affected individuals and their families.

In our case, we could not confirm whether the variant was *de novo*, as the patient’s father, who died at the age of 50 without presenting neurological symptoms, could not be tested. Although the absence of symptoms in mid-adulthood makes paternal inheritance unlikely, it cannot be entirely ruled out given the inherited cases reported by Richards et al. (2015); Brooker et al. (2025), and the variable expressivity that has been reported in *NUS1*-related conditions.

### 4.8 Molecular spectrum of *NUS1* variants

A wide spectrum of genetic variants has been reported in *NUS1*, including whole-gene and partial deletions, as well as single nucleotide variants (SNVs) encompassing intronic, splice site, and coding variants. In terms of the molecular effect, the majority of *NUS1* variants are truncating, although several missense variants have also been documented (Park et al., 2014; Yu et al., 2021; Riboldi et al., 2022; Ji et al., 2023; Shen et al., 2024).

The *NUS1* gene encodes a protein with four key functional domains: TM1, TM2, TM3 and the cis-prenlytransferase (cis-PTase) domain (Riboldi et al., 2022; Best et al., 2025). The C-terminal region, which includes the highly conserved cis-PTase domain, is essential for mediating protein–protein interactions required for proper enzymatic function (Den et al., 2019; Hamdan et al., 2017). Truncating variants within this region have been hypothesized to result in a milder clinical phenotype, as they are located beyond the typical threshold for triggering nonsense-mediated decay (NMD) (the “<−50 bp rule”) (Den et al., 2019). In contrast, upstream truncating variants are more likely to result in complete transcript degradation via NMD and may therefore be associated with more severe phenotypes, consistent with the proposed haploinsufficiency mechanism underlying *NUS1*-related disorders (Yu et al., 2021; Li et al., 2024; Den et al., 2019; Landais et al., 2025). However, clinical observations to date do not consistently support this hypothesis, as individuals with C-terminal truncating variants do not always exhibit milder phenotypes compared to those with more proximal variants (Hu et al., 2023; Den et al., 2019; Brooker et al., 2025), as reported in the literature and as observed in our review. Therefore, while domain-based hypotheses remain biologically plausible, the current evidence is insufficient to establish a clear genotype–phenotype correlation, and additional well-characterized cases will be necessary to better delineate these relationships.

Emerging evidence suggests that some truncated transcripts only undergo partial degradation by NMD, potentially allowing the production of aberrant or partially functional proteins (Hu et al., 2023; Den et al., 2019). This phenomenon may be explained by alternative mechanisms such as nonsense-associated altered splicing (NAS) (Lambert et al., 2020). This incomplete degradation could contribute to the phenotypic variability observed among individuals with different *NUS1* variants. Further mRNA and protein expression studies are needed to determine whether partially functional proteins are produced and how they may influence the observed clinical outcomes. Additionally, the presence of modifying variants in other genes, particularly those within the *NUS1*-associated protein-protein interaction network, as described by Ding et al. (2025), may also play a role in modulating disease expression.

In our case, the patient carries a truncating variant in exon 1, located between the TM2 and TM3 domains (Figure 2). Due to its early position in the coding sequence, the transcript is likely subject to NMD degradation. However, further studies are needed to better improve understanding of the mechanisms through which this variant could contribute to the patient’s clinical presentation.

A broad phenotypic spectrum has been described among individuals with *NUS1*, even among those harboring identical variants. The molecular basis underlying the broad phenotypic spectrum associated with *NUS1* variants remains unclear. It is not yet established whether specific variant types or locations consistently correlate with particular clinical features. Further research is required to elucidate potential genotype-phenotype correlations, and to determine whether certain variants may predispose individuals to specific clinical presentations, degrees of severity, or variability. Understanding these relationships is crucial to effectively counsel patients and their families regarding prognosis, management, and reproductive options.

## Data Availability

The original contributions presented in the study are publicly available. This data can be found in the European Nucleotide Archive (ENA) repository with the accession numbers PRJEB104382 and ERP185676.
