# Supplementary material for: Expanding the spectrum of NUS1-related progressive myoclonic epilepsy: a novel variant and exploratory use of metformin
Source: Front Genet. 2025 Dec 18;16:1665623. doi: 10.3389/fgene.2025.1665623 (PMC12755855; doi:10.3389/fgene.2025.1665623)
Supplement: Supplementary file 1 [file Table1.docx]

Supplementary Material

# Supplementary Data

Genes analyzed associated with epilepsy:

*AAAS, AARS, AARS2, AASS, ABAT, ABCA1, ABCA12, ABCA2, ABCA4, ABCA5, ABCA7, ABCB11, ABCB4, ABCB6, ABCB7, ABCC6, ABCC8, ABCC9, ABCD1, ABCD4, ABHD12, ABHD5, ACADM, ACADS, ACADSB, ACAN, ACAT1, ACAT2, ACBD5, ACD, ACER3, ACO2, ACOX1, ACOX2, ACP5, ACSF3, ACSL4, ACTA1, ACTA2, ACTB, ACTG1, ACTL6A, ACTL6B, ACTN2, ACVR1, ACVRL1, ACY1, ADA2, ADAM22, ADAM9, ADAMTS10, ADAMTS13, ADAMTS2, ADAMTS3, ADAMTSL2, ADAR, ADARB1, ADAT3, ADCY3, ADCY5, ADCY6, ADD3, ADGRG1, ADGRV1, ADH1B, ADH1C, ADK, ADNP, ADPRHL2, ADRA2B, ADSL, ADSSL1, AFF2, AFF4, AFG3L2, AGA, AGBL5, AGGF1, AGL, AGPAT2, AGRN, AGTPBP1, AGTR2, AHCY, AHDC1, AHI1, AHR, AHSG, AIFM1, AIMP1, AIMP2, AIP, AIPL1, AIRE, AK9, AKAP9, AKT1, AKT2, AKT3, ALAD, ALDH18A1, ALDH3A2, ALDH4A1, ALDH5A1, ALDH6A1, ALDH7A1, ALDOA, ALDOB, ALG1, ALG11, ALG12, ALG13, ALG14, ALG2, ALG3, ALG6, ALG9, ALKBH8, ALMS1, ALOX12B, ALOXE3, ALPL, ALS2, ALX1, ALX3, ALX4, AMACR, AMER1, AMMECR1, AMPD1, AMPD2, AMT, ANAPC1, ANG, ANGPTL6, ANK1, ANK2, ANK3, ANKH, ANKLE2, ANKRD11, ANO10, ANO3, ANO5, ANOS1, ANTXR1, ANXA11, AP1B1, AP1S1, AP1S2, AP2M1, AP2S1, AP3B1, AP3B2, AP3D1, AP4B1, AP4E1, AP4M1, AP4S1, AP5Z1, APC, APC2, APOB, APOE, APOL2, APOL4, APOPT1, APP, APTX, AQP2, AR, ARCN1, ARF1, ARFGEF2, ARG1, ARHGAP31, ARHGDIA, ARHGEF18, ARHGEF2, ARHGEF6, ARHGEF9, ARID1A, ARID1B, ARID2, ARL13B, ARL2BP, ARL3, ARL6, ARL6IP1, ARL6IP6, ARMC5, ARMC9, ARNT2, ARSA, ARSG, ARSI, ARV1, ARVCF, ARX, ASAH1, ASCC1, ASCL1, ASH1L, ASL, ASNS, ASPA, ASPM, ASS1, ASXL1, ASXL2, ASXL3, ATAD1, ATAD3A, ATCAY, ATF6, ATIC, ATL1, ATL3, ATM, ATN1, ATP10A, ATP13A2, ATP1A1, ATP1A2, ATP1A3, ATP2A2, ATP2B3, ATP5F1A, ATP5F1D, ATP5F1E, ATP5MD, ATP6, ATP6AP1, ATP6AP2, ATP6V0A2, ATP6V1A, ATP6V1B2, ATP6V1E1, ATP7A, ATP7B, ATP8, ATP8A2, ATP8B1, ATPAF2, ATR, ATRIP, ATRX, ATXN1, ATXN10, ATXN2, ATXN3, ATXN7, ATXN8OS, AUH, AUTS2, AVP, AVPR2, B3GALNT2, B3GALT6, B3GLCT, B4GALNT1, B4GAT1, B9D1, B9D2, BAG3, BAP1, BAZ1B, BBIP1, BBS1, BBS10, BBS12, BBS2, BBS4, BBS5, BBS7, BBS9, BCAP31, BCAT2, BCKDHA, BCKDHB, BCKDK, BCL10, BCL11A, BCL11B, BCOR, BCORL1, BCR, BCS1L, BDNF, BEAN1, BEST1, BICD2, BIN1, BLM, BMP2, BMP4, BMPR1A, BOLA3, BPTF, BRAF, BRAT1, BRCA1, BRCA2, BRF1, BRIP1, BRPF1, BRSK2, BRWD3, BSCL2, BSND, BTD, BUB1, BUB1B, BUB3, BVES, C11orf95, C12orf4, C12orf57, C12orf65, C16orf62, C19orf12, C19orf70, C21orf2, C2CD3, C2orf71, C4A, C5orf42, C7orf43, C8orf37, C9orf72, CA2, CA4, CA8, CABP4, CACNA1A, CACNA1B, CACNA1C, CACNA1D, CACNA1E, CACNA1F, CACNA1G, CACNA1H, CACNA1S, CACNA2D2, CACNA2D4, CACNB4, CACNG2, CAD, CALM1, CALM2, CALM3, CAMK2A, CAMK2B, CAMKMT, CAMTA1, CANT1, CAPN1, CAPN3, CARS, CARS2, CASK, CASP10, CASQ2, CASR, CAT, CAV1, CAV3, CAVIN1, CBL, CBS, CC2D1A, CC2D2A, CCBE1, CCDC115, CCDC141, CCDC22, CCDC28B, CCDC47, CCDC88A, CCDC88C, CCM2, CCND2, CCNF, CCNQ, CCR1, CCT5, CD109, CD28, CD59, CD96, CDC42, CDC45, CDC6, CDC73, CDCA7, CDH11, CDH15, CDH2, CDH23, CDHR1, CDK10, CDK13, CDK19, CDK5, CDK5RAP2, CDK6, CDK8, CDKL5, CDKN1A, CDKN1B, CDKN1C, CDKN2B, CDKN2C, CDON, CDT1, CENPE, CENPJ, CEP104, CEP120, CEP126, CEP135, CEP152, CEP164, CEP19, CEP250, CEP290, CEP41, CEP57, CEP63, CEP78, CEP83, CEP85L, CERKL, CERS1, CFAP43, CFH, CFHR1, CFHR3, CFL2, CHAMP1, CHAT, CHCHD10, CHCHD2, CHD1, CHD2, CHD3, CHD4, CHD7, CHD8, CHI3L1, CHKB, CHMP1A, CHMP2B, CHN1, CHP1, CHRNA1, CHRNA2, CHRNA4, CHRNA7, CHRNB1, CHRNB2, CHRND, CHRNE, CHRNG, CHST14, CHST3, CHST6, CHSY1, CIB2, CIC, CIITA, CISD2, CIT, CIZ1, CKAP2L, CLCC1, CLCF1, CLCN2, CLCN4, CLCN7, CLCNKA, CLCNKB, CLDN10, CLDN16, CLEC7A, CLIC2, CLIP1, CLIP2, CLN3, CLN5, CLN6, CLN8, CLP1, CLPB, CLPP, CLRN1, CLTC, CLTCL1, CNGA1, CNGA3, CNGB1, CNGB3, CNKSR2, CNNM2, CNNM4, CNOT1, CNOT3, CNP, CNPY3, CNTN1, CNTN2, CNTNAP1, CNTNAP2, COA7, COASY, COG1, COG2, COG4, COG5, COG6, COG7, COG8, COL10A1, COL12A1, COL13A1, COL17A1, COL18A1, COL1A1, COL1A2, COL2A1, COL3A1, COL4A1, COL4A2, COL4A3BP, COL6A1, COL6A2, COL6A3, COL7A1, COL8A2, COL9A1, COL9A2, COL9A3, COLEC10, COLEC11, COLGALT1, COLQ, COMP, COMT, COPB2, COQ2, COQ4, COQ5, COQ6, COQ8A, COQ9, CORIN, CORO1A, COX10, COX15, COX20, COX3, COX4I1, COX5A, COX6A1, COX7B, COX8A, CP, CPA6, CPLX1, CPOX, CPS1, CPT1A, CPT1C, CPT2, CRADD, CRAT, CRB1, CRB2, CRBN, CREBBP, CRIPT, CRKL, CRLF1, CRPPA, CRX, CRY1, CRYAB, CRYGC, CSF1R, CSGALNACT1, CSNK1D, CSNK2A1, CSNK2B, CSPP1, CST6, CSTB, CTBP1, CTC1, CTCF, CTDP1, CTH, CTLA4, CTNNA2, CTNNB1, CTNND2, CTNS, CTSA, CTSD, CTSF, CTSH, CTU2, CUL4B, CUX1, CUX2, CWC27, CWF19L1, CXCR4, CXorf56, CYB5A, CYB5R3, CYFIP2, CYP11B1, CYP11B2, CYP24A1, CYP26C1, CYP27A1, CYP27B1, CYP2R1, CYP2U1, CYP7B1, CYTB, D2HGDH, DAB1, DACT1, DAG1, DALRD3, DAO, DAOA, DARS, DARS2, DBH, DBT, DCAF17, DCAF8, DCC, DCHS1, DCPS, DCTN1, DCX, DDB2, DDC, DDHD1, DDHD2, DDOST, DDRGK1, DDX11, DDX3X, DDX59, DDX6, DEAF1, DEGS1, DENND5A, DEPDC5, DES, DGCR2, DGCR6, DGCR8, DGUOK, DHCR24, DHCR7, DHDDS, DHFR, DHH, DHPS, DHTKD1, DHX16, DHX30, DHX37, DHX38, DIAPH1, DIP2B, DIS3L2, DISP1, DKC1, DKK1, DLAT, DLD, DLG3, DLG4, DLK1, DLL1, DLL3, DLL4, DLST, DLX5, DMD, DMPK, DMXL2, DNA2, DNAJB2, DNAJB6, DNAJC12, DNAJC13, DNAJC19, DNAJC21, DNAJC3, DNAJC5, DNAJC6, DNAL4, DNASE1, DNASE1L3, DNM1, DNM1L, DNM2, DNMT1, DNMT3A, DNMT3B, DOCK3, DOCK6, DOCK7, DOCK8, DOK7, DOLK, DONSON, DPAGT1, DPF2, DPH1, DPM1, DPM2, DPM3, DPP6, DPYD, DPYS, DRAM2, DRD2, DRD3, DRD4, DRD5, DSG4, DST, DSTYK, DUOX2, DUOXA2, DUSP6, DVL1, DVL3, DYM, DYNC1H1, DYNC1I2, DYNC2LI1, DYRK1A, DYSF, DZIP1L, EARS2, EBF3, EBP, ECE1, ECHS1, ECM1, EDC3, EDN1, EDN3, EDNRA, EDNRB, EED, EEF1A2, EEF2, EFEMP2, EFHC1, EFL1, EFNB1, EFTUD2, EGF, EGR2, EHMT1, EIF2AK2, EIF2AK3, EIF2B1, EIF2B2, EIF2B3, EIF2B4, EIF2B5, EIF2S3, EIF3F, EIF4G1, ELMO2, ELN, ELOVL1, ELOVL4, ELOVL5, ELP1, ELP2, EMC1, EMD, EMG1, EML1, ENG, ENPP1, ENTPD1, EOGT, EP300, EPAS1, EPB41L1, EPCAM, EPG5, EPHA4, EPM2A, EPRS, ERAP1, ERBB3, ERBB4, ERCC1, ERCC2, ERCC3, ERCC4, ERCC5, ERCC6, ERCC8, ERF, ERLIN1, ERLIN2, ERMARD, ESCO2, ESPN, ESR1, ESS2, ETHE1, EVC, EVC2, EXOC6B, EXOC7, EXOC8, EXOSC2, EXOSC3, EXOSC9, EXT1, EXT2, EXTL3, EYA1, EYS, EZH2, EZR, F8, FA2H, FADD, FAM111A, FAM126A, FAM149B1, FAM161A, FAM50A, FAN1, FANCA, FANCB, FANCC, FANCD2, FANCE, FANCF, FANCG, FANCI, FANCL, FANCM, FAR1, FARS2, FARSB, FAS, FASLG, FASTKD2, FAT2, FAT4, FBLN1, FBLN5, FBN1, FBP1, FBXL4, FBXO11, FBXO31, FBXO38, FBXO7, FBXW11, FCGR2A, FCGR2B, FDFT1, FDX2, FDXR, FEZF1, FGD1, FGD4, FGF12, FGF14, FGF17, FGF8, FGFR1, FGFR2, FGFR3, FGFRL1, FH, FHL1, FIBP, FIG4, FIP1L1, FITM2, FKBP10, FKRP, FKTN, FLCN, FLI1, FLII, FLNA, FLNB, FLNC, FLRT1, FLRT3, FLT1, FLT4, FLVCR1, FLVCR2, FMN2, FMO3, FMR1, FN1, FOLR1, FOS, FOXA2, FOXC2, FOXE1, FOXE3, FOXG1, FOXH1, FOXI1, FOXP1, FOXP2, FOXP3, FOXRED1, FRAS1, FREM2, FRG1, FRMD4A, FRMPD4, FRRS1L, FSCN2, FTCD, FTL, FTO, FTSJ1, FUCA1, FUK, FUS, FUT8, FUZ, FXN, FXR1, FXYD2, FZD2, GAA, GABBR2, GABRA1, GABRA2, GABRA3, GABRA5, GABRB1, GABRB2, GABRB3, GABRD, GABRG2, GAD1, GAL, GALC, GALE, GALK1, GALNT2, GALT, GAMT, GAN, GARS, GAS1, GATA1, GATA3, GATA4, GATA6, GATAD2B, GATM, GBA, GBA2, GBE1, GCDH, GCH1, GCK, GCLC, GCM2, GCSH, GDAP1, GDAP2, GDF2, GDF6, GDI1, GDNF, GEMIN4, GFAP, GFER, GFM1, GFM2, GFPT1, GGT1, GHR, GIGYF2, GJA1, GJA5, GJA8, GJB1, GJB2, GJB3, GJB4, GJB6, GJC2, GK, GLA, GLB1, GLDC, GLE1, GLI1, GLI2, GLI3, GLRA1, GLRB, GLRX5, GLS, GLT8D1, GLUD1, GLUD2, GLUL, GLYCTK, GM2A, GMNN, GMPPA, GMPPB, GNA11, GNAI3, GNAL, GNAO1, GNAQ, GNAS, GNAT2, GNB1, GNB3, GNB4, GNB5, GNE, GNPAT, GNPTAB, GNPTG, GNRH1, GNRHR, GNS, GORAB, GOSR2, GOT2, GP1BA, GP1BB, GP9, GPAA1, GPC1, GPC3, GPC4, GPC6, GPHN, GPI, GPR101, GPR143, GPR161, GPR35, GPR88, GPSM2, GPT2, GRHL2, GRIA2, GRIA3, GRIA4, GRID2, GRIK2, GRIN1, GRIN2A, GRIN2B, GRIN2D, GRIP1, GRM1, GRM7, GRN, GSN, GSS, GSX2, GTF2E2, GTF2H5, GTF2I, GTF2IRD1, GTPBP2, GTPBP3, GUCA1A, GUCA1B, GUCY1A1, GUCY2D, GUF1, GUSB, GYG1, GYS1, GYS2, H19, HAAO, HACD1, HACE1, HADH, HADHA, HADHB, HAL, HARS, HAX1, HBA1, HBA2, HBB, HCCS, HCFC1, HCN1, HCRT, HDAC4, HDAC6, HDAC8, HDC, HECW2, HELLS, HEPACAM, HERC1, HERC2, HES7, HESX1, HEXA, HEXB, HFE, HGSNAT, HHAT, HIBCH, HIC1, HIKESHI, HINT1, HIRA, HIST1H1E, HIVEP2, HK1, HLA-A, HLA-B, HLA-DPA1, HLA-DPB1, HLA-DQB1, HLA-DRB1, HLCS, HMBS, HMGA2, HMGB3, HMGCL, HMGCS2, HNF1A, HNF1B, HNF4A, HNMT, HNRNPA1, HNRNPA2B1, HNRNPDL, HNRNPH2, HNRNPK, HNRNPU, HOXA1, HOXA2, HPCA, HPD, HPDL, HPGD, HPRT1, HPS6, HPSE2, HRAS, HS6ST1, HS6ST2, HSD11B2, HSD17B10, HSD17B4, HSPB1, HSPB3, HSPB8, HSPD1, HSPG2, HTR2A, HTRA1, HTRA2, HTT, HUWE1, HYLS1, IARS, IARS2, IBA57, ICK, IDH1, IDH2, IDH3A, IDH3B, IDS, IDUA, IER3IP1, IFIH1, IFNG, IFRD1, IFT140, IFT172, IFT27, IFT74, IFT88, IGBP1, IGF1, IGF1R, IGF2, IGHMBP2, IKBKG, IKZF1, IL10, IL12A, IL12B, IL12RB1, IL17F, IL17RA, IL17RC, IL17RD, IL1RAPL1, IL23R, IL6, IMPA1, IMPDH1, IMPG2, INF2, INPP5E, INPP5K, INS, INSR, INTS1, INTS8, IQCB1, IQSEC1, IQSEC2, IRAK1, IREB2, IRF2BP2, IRF2BPL, IRF3, IRF5, IRF6, ISCA1, ISCA2, ISG15, ISPD, ITGA2, ITGA2B, ITGA7, ITGB3, ITGB6, ITM2B, ITPA, ITPR1, ITPR3, IVD, IYD, JAG1, JAK2, JAM2, JAM3, JMJD1C, JPH1, JPH3, JRK, KANK1, KANSL1, KARS, KAT6A, KAT6B, KAT8, KATNB1, KBTBD13, KCNA1, KCNA2, KCNA4, KCNAB2, KCNB1, KCNC1, KCNC3, KCND3, KCNE1, KCNE2, KCNE5, KCNH1, KCNH2, KCNJ1, KCNJ10, KCNJ11, KCNJ13, KCNJ18, KCNJ2, KCNJ5, KCNJ6, KCNJ8, KCNK4, KCNK9, KCNMA1, KCNN3, KCNQ1, KCNQ2, KCNQ3, KCNQ5, KCNT1, KCNT2, KCNV2, KCTD17, KCTD7, KDM1A, KDM3B, KDM5B, KDM5C, KDM6A, KDM6B, KDSR, KIAA0556, KIAA0586, KIAA0753, KIAA1109, KIAA1549, KIDINS220, KIF11, KIF14, KIF15, KIF1A, KIF1B, KIF1BP, KIF1C, KIF2A, KIF4A, KIF5A, KIF5C, KIF7, KIRREL3, KISS1, KISS1R, KIT, KIZ, KLC2, KLF13, KLHL15, KLHL40, KLHL41, KLHL7, KLHL9, KLLN, KLRC4, KMT2A, KMT2B, KMT2C, KMT2D, KMT2E, KMT5B, KNL1, KNSTRN, KPTN, KRAS, KRIT1, KRT12, KRT3, KRT81, KRT83, KRT86, KY, KYNU, L1CAM, L2HGDH, LAGE3, LAMA1, LAMA2, LAMA3, LAMB1, LAMB2, LAMB3, LAMC2, LAMC3, LAMP2, LARGE1, LARP7, LARS, LARS2, LAS1L, LBR, LCA5, LDB3, LDHD, LEMD3, LEP, LEPR, LETM1, LFNG, LGI1, LGI4, LHCGR, LHX1, LHX3, LHX4, LIAS, LIFR, LIG4, LIMK1, LINGO1, LINS1, LIPT1, LIPT2, LITAF, LMAN2L, LMBR1, LMBRD1, LMNA, LMNB1, LMNB2, LMOD3, LMX1B, LNPK, LONP1, LPIN1, LRAT, LRIG2, LRMDA, LRP12, LRP2, LRP4, LRP5, LRPPRC, LRRC32, LRRK2, LRSAM1, LSS, LTBP2, LTBP3, LTBP4, LYRM7, LYST, LZTFL1, MAB21L1, MAB21L2, MACF1, MAD2L2, MADD, MAF, MAFB, MAG, MAGEL2, MAGT1, MAK, MAN1B1, MAN2B1, MANBA, MAOA, MAP1B, MAP2K1, MAP2K2, MAP3K20, MAP3K7, MAPK1, MAPK10, MAPK8IP3, MAPRE2, MAPT, MARCH6, MARS, MARS2, MASP1, MAST1, MAT1A, MATN3, MATR3, MAX, MBD5, MBOAT7, MBTPS1, MBTPS2, MC1R, MC2R, MC4R, MCCC1, MCCC2, MCM3AP, MCOLN1, MCPH1, MCTP2, MDH1, MDH2, MECOM, MECP2, MECR, MED12, MED12L, MED13, MED13L, MED17, MED23, MED25, MEF2C, MEFV, MEGF10, MEGF8, MEIS2, MEN1, MERTK, MESP2, METTL23, METTL5, MFF, MFN2, MFRP, MFSD2A, MFSD8, MGAT2, MGME1, MGP, MICAL1, MICU1, MID2, MIEF2, MIPEP, MKKS, MKRN3, MKS1, MLC1, MLH1, MLH3, MLX, MLXIPL, MLYCD, MMAA, MMACHC, MMADHC, MME, MMEL1, MMP1, MMP13, MMP14, MMP2, MN1, MOCS1, MOCS2, MOG, MOGS, MORC2, MPC1, MPDU1, MPDZ, MPL, MPLKIP, MPV17, MPZ, MRAP, MRE11, MRM2, MRPL12, MRPS16, MRPS2, MRPS22, MRPS25, MRPS34, MSH2, MSH6, MSL3, MSMO1, MST1, MSTO1, MSX1, MSX2, MT-CO1, MT-CO2, MT-ND1, MT-TL2, MTFMT, MTHFD1, MTHFR, MTHFS, MTM1, MTMR14, MTO1, MTOR, MTPAP, MTR, MTRR, MTTP, MUSK, MUT, MVK, MYBPC1, MYCN, MYF5, MYF6, MYH14, MYH2, MYH3, MYH7, MYL1, MYL2, MYMK, MYO1H, MYO5A, MYO7A, MYO9A, MYOD1, MYORG, MYOT, MYPN, MYRF, MYSM1, MYT1L, NAA10, NAA15, NABP1, NACC1, NADK2, NAGA, NAGLU, NAGS, NALCN, NANS, NARS, NARS2, NAT8L, NAXD, NAXE, NBAS, NBN, NCAPD2, NCAPD3, NCAPH, ND2, ND3, ND4, ND4L, ND5, ND6, NDE1, NDN, NDNF, NDP, NDRG1, NDST1, NDUFA1, NDUFA10, NDUFA11, NDUFA12, NDUFA13, NDUFA2, NDUFA4, NDUFA6, NDUFA9, NDUFAF1, NDUFAF2, NDUFAF3, NDUFAF4, NDUFAF5, NDUFAF6, NDUFAF8, NDUFB10, NDUFB11, NDUFB3, NDUFB8, NDUFB9, NDUFS1, NDUFS2, NDUFS3, NDUFS4, NDUFS6, NDUFS7, NDUFS8, NDUFV1, NDUFV2, NEB, NECAP1, NECTIN1, NEDD4L, NEFH, NEFL, NEK1, NEK2, NEK9, NELFA, NEMF, NEU1, NEUROD2, NEXMIF, NF1, NF2, NFASC, NFE2L2, NFIA, NFIB, NFIX, NFKB2, NFU1, NGF, NGLY1, NHLRC1, NHLRC2, NHP2, NHS, NIN, NIPA1, NIPBL, NKAP, NKX2-1, NKX2-5, NKX6-2, NLGN1, NLGN3, NLGN4X, NLRP1, NLRP3, NMNAT1, NNT, NOD2, NODAL, NOG, NOL3, NONO, NOP10, NOP56, NOS1AP, NOS3, NOTCH1, NOTCH3, NOVA2, NPAP1, NPC1, NPC2, NPHP1, NPHP3, NPHP4, NPM1, NPRL2, NPRL3, NR1H4, NR2E3, NR2F1, NR3C1, NR4A2, NRAS, NRL, NRROS, NRTN, NRXN1, NSD1, NSD2, NSDHL, NSMF, NSUN2, NT5C2, NTN1, NTNG1, NTNG2, NTRK1, NTRK2, NUBPL, NUMA1, NUP107, NUP133, NUP214, NUP62, NUP88, NUS1, NXN, OAT, OCA2, OCLN, OCRL, ODC1, OFD1, OGDH, OGT, OPA1, OPA3, OPHN1, OPN1LW, OPN1MW, OPN1SW, OPTN, ORAI1, ORC1, ORC4, ORC6, OSGEP, OSTM1, OTC, OTOG, OTUD6B, OTX2, OVOL2, OXR1, P2RY11, P4HTM, PABPN1, PACS1, PACS2, PAFAH1B1, PAH, PAK1, PAK3, PALB2, PAM16, PANK2, PARK7, PARN, PARS2, PAX1, PAX2, PAX3, PAX6, PAX7, PAX8, PC, PCBD1, PCCA, PCCB, PCDH12, PCDH15, PCDH19, PCGF2, PCK1, PCLO, PCNA, PCNT, PCSK1, PCYT1A, PCYT2, PDCD1, PDCD10, PDE10A, PDE11A, PDE4D, PDE6A, PDE6B, PDE6C, PDE6D, PDE6G, PDE6H, PDE8B, PDGFB, PDGFRB, PDHA1, PDHB, PDHX, PDK3, PDP1, PDSS1, PDSS2, PDX1, PDXK, PDYN, PDZD7, PEPD, PER2, PER3, PERP, PET100, PET117, PEX1, PEX10, PEX11B, PEX12, PEX13, PEX14, PEX16, PEX19, PEX2, PEX26, PEX3, PEX5, PEX6, PEX7, PFN1, PGAP1, PGAP2, PGAP3, PGK1, PGM3, PHACTR1, PHC1, PHF21A, PHF6, PHF8, PHGDH, PHIP, PHKA2, PHKG2, PHOX2B, PHYH, PI4KA, PIBF1, PIEZO2, PIGA, PIGB, PIGC, PIGG, PIGH, PIGK, PIGL, PIGM, PIGN, PIGO, PIGP, PIGQ, PIGS, PIGT, PIGU, PIGV, PIGW, PIGY, PIK3C2A, PIK3CA, PIK3CD, PIK3R2, PIK3R5, PIKFYVE, PINK1, PISD, PITPNM3, PITX3, PKDCC, PKHD1, PLA2G6, PLAA, PLAG1, PLAGL1, PLCB1, PLCB4, PLCD1, PLD3, PLEC, PLEKHG2, PLEKHG4, PLEKHG5, PLK4, PLOD1, PLP1, PLPBP, PLXND1, PML, PMM2, PMP2, PMP22, PMPCA, PMPCB, PMS1, PMS2, PNKD, PNKP, PNP, PNPLA2, PNPLA6, PNPLA8, PNPO, PNPT1, POC1A, POC1B, PODXL, POGZ, POLA1, POLE, POLG, POLG2, POLH, POLR1C, POLR1D, POLR2A, POLR3A, POLR3B, POMC, POMGNT1, POMGNT2, POMK, POMT1, POMT2, PON1, PON2, PON3, POPDC3, POR, PORCN, POU1F1, POU2AF1, POU3F3, POU3F4, PPA2, PPARG, PPARGC1A, PPM1B, PPM1D, PPOX, PPP1CB, PPP1R12A, PPP1R15B, PPP2CA, PPP2R1A, PPP2R2B, PPP2R5D, PPP3CA, PPT1, PQBP1, PRCD, PRDM16, PRDM5, PRDM8, PRDX1, PREPL, PRF1, PRICKLE1, PRKACA, PRKAG2, PRKAR1A, PRKAR1B, PRKCD, PRKCG, PRKDC, PRKN, PRKRA, PRMT7, PRNP, PROC, PRODH, PROK2, PROKR2, PROM1, PROP1, PRPF3, PRPF31, PRPF4, PRPF6, PRPF8, PRPH, PRPH2, PRPS1, PRRT2, PRSS12, PRTN3, PRUNE1, PRX, PSAP, PSAT1, PSEN1, PSEN2, PSMB8, PSMD12, PSPH, PTCD3, PTCH1, PTCH2, PTCHD1, PTDSS1, PTEN, PTF1A, PTH, PTH1R, PTPN11, PTPN22, PTPN23, PTRH2, PTS, PUF60, PUM1, PURA, PUS1, PUS3, PUS7, PYCR1, PYCR2, PYGL, PYROXD1, QARS, QDPR, QRICH1, RAB11A, RAB11B, RAB18, RAB23, RAB27A, RAB28, RAB39B, RAB3GAP1, RAB3GAP2, RAB7A, RAC1, RAC3, RAD21, RAD50, RAD51, RAD51C, RAF1, RAI1, RALGAPA1, RANBP2, RAPGEF2, RAPSN, RARA, RARB, RARS, RARS2, RASGRP1, RAX2, RB1, RBBP8, RBM10, RBM12, RBM28, RBM8A, RBMX, RBP3, RBPJ, RD3, RDH11, RDH12, RDH5, RECQL4, REEP1, REEP2, REEP6, RELA, RELN, REPS1, RERE, RET, RETREG1, REV3L, RFC1, RFC2, RFT1, RFWD3, RFX5, RFXANK, RFXAP, RGR, RGS9, RGS9BP, RHO, RHOBTB2, RIC1, RIMS1, RIMS2, RIPK4, RIPPLY2, RIT1, RLBP1, RLIM, RMND1, RMRP, RNASEH1, RNASEH2A, RNASEH2B, RNASEH2C, RNASET2, RNF113A, RNF125, RNF13, RNF135, RNF168, RNF170, RNF213, RNF216, RNR1, RNU4ATAC, ROBO1, ROBO3, ROGDI, ROM1, ROR2, RORA, RORB, RP1, RP1L1, RP2, RP9, RPE65, RPGR, RPGRIP1, RPGRIP1L, RPIA, RPL10, RPS19, RPS20, RPS23, RPS6KA3, RREB1, RRM2B, RSPRY1, RSRC1, RTEL1, RTL1, RTN2, RTN4IP1, RTN4R, RTTN, RUBCN, RUNX1, RUNX2, RUSC2, RXYLT1, RYR1, RYR2, SACS, SAG, SALL1, SALL4, SAMD12, SAMD9, SAMD9L, SAMHD1, SAR1B, SARDH, SARS, SASS6, SATB2, SBDS, SBF1, SBF2, SC5D, SCAPER, SCARB2, SCN10A, SCN11A, SCN1A, SCN1B, SCN2A, SCN3A, SCN4A, SCN4B, SCN5A, SCN8A, SCN9A, SCO2, SCP2, SCYL1, SCYL2, SDCCAG8, SDHA, SDHAF1, SDHAF2, SDHB, SDHC, SDHD, SEC23B, SEC24C, SEC31A, SELENOI, SELENON, SEMA3A, SEMA3C, SEMA3D, SEMA3E, SEMA4A, SEMA5A, SEMA6B, SEPSECS, set-09, SERAC1, SERPINI1, SET, SETBP1, SETD1A, SETD1B, SETD2, SETD5, SETX, SFXN4, SGCA, SGCB, SGCD, SGCE, SGCG, SGPL1, SGSH, SH3BP2, SH3KBP1, SH3TC2, SHANK3, SHH, SHMT2, SHOC2, SHROOM4, SIGMAR1, SIK1, SIK3, SIL1, SIM1, SIN3A, SIX3, SKI, SKIV2L, SLC12A1, SLC12A2, SLC12A3, SLC12A5, SLC12A6, SLC13A5, SLC16A1, SLC16A2, SLC17A5, SLC18A2, SLC18A3, SLC19A2, SLC19A3, SLC1A1, SLC1A2, SLC1A3, SLC1A4, SLC20A2, SLC22A5, SLC24A5, SLC25A1, SLC25A10, SLC25A11, SLC25A12, SLC25A13, SLC25A15, SLC25A19, SLC25A20, SLC25A21, SLC25A22, SLC25A24, SLC25A4, SLC25A42, SLC25A46, SLC26A4, SLC29A3, SLC2A1, SLC2A10, SLC2A3, SLC30A10, SLC30A9, SLC33A1, SLC34A3, SLC35A1, SLC35A2, SLC35A3, SLC35C1, SLC36A2, SLC39A13, SLC39A14, SLC39A4, SLC39A8, SLC3A1, SLC44A1, SLC45A1, SLC45A2, SLC46A1, SLC4A4, SLC52A2, SLC52A3, SLC5A2, SLC5A5, SLC5A6, SLC5A7, SLC6A1, SLC6A17, SLC6A19, SLC6A20, SLC6A3, SLC6A4, SLC6A5, SLC6A8, SLC6A9, SLC7A14, SLC7A7, SLC9A1, SLC9A6, SLC9A7, SLCO2A1, SLITRK1, SLITRK6, SLX4, SMAD4, SMARCA2, SMARCA4, SMARCAL1, SMARCB1, SMARCC2, SMARCD1, SMARCE1, SMC1A, SMC3, SMCHD1, SMG9, SMN1, SMN2, SMO, SMOC1, SMPD1, SMPD4, SMS, SNAI2, SNAP25, SNAP29, SNCA, SNCAIP, SNCB, SNIP1, SNRNP200, SNRPB, SNRPN, SNTA1, SNX10, SNX14, SOBP, SOD1, SON, SORD, SORL1, SOST, SOX10, SOX11, SOX2, SOX3, SOX4, SOX5, SP110, SPART, SPAST, SPATA5, SPATA7, SPECC1L, SPEG, SPG11, SPG21, SPG7, SPIB, SPINK5, SPOP, SPP1, SPR, SPRED1, SPRY4, SPTAN1, SPTBN2, SPTBN4, SPTLC1, SQSTM1, SRCAP, SRD5A3, SREBF1, SRP54, SRPX2, SRSF2, SRY, SSR4, ST14, ST3GAL3, ST3GAL5, STAC3, STAG1, STAG2, STAMBP, STAR, STARD7, STAT2, STAT3, STAT4, STAT5B, STIL, STIM1, STN1, STOX1, STRA6, STRADA, STS, STT3A, STT3B, STUB1, STX11, STX16, STX1B, STXBP1, SUCLA2, SUCLG1, SUFU, SUMF1, SUOX, SURF1, SUZ12, SVBP, SYN1, SYN2, SYNE1, SYNE2, SYNGAP1, SYNJ1, SYP, SYT1, SYT14, SYT2, SZT2, TAB2, TAC3, TACO1, TACR3, TACSTD2, TAF1, TAF13, TAF15, TAF2, TAF6, TANC2, TANGO2, TAOK1, TARDBP, TARS, TASP1, TAT, TAZ, TBC1D20, TBC1D23, TBC1D24, TBC1D7, TBCD, TBCE, TBCK, TBK1, TBL1X, TBL1XR1, TBL2, TBP, TBR1, TBX1, TBX19, TBX2, TBX4, TCAP, TCF12, TCF20, TCF4, TCIRG1, TCN2, TCOF1, TCTN1, TCTN2, TCTN3, TDGF1, TDP1, TDP2, TECPR2, TECR, TELO2, TENM3, TENM4, TERT, TET2, TET3, TFAP2A, TFAP2B, TFG, TG, TGDS, TGFB1, TGFB2, TGFB3, TGFBI, TGFBR1, TGFBR2, TGFBR3, TGIF1, TGM6, TH, THAP1, THG1L, THOC2, THOC6, THPO, THRA, THRB, THSD1, TIA1, TICAM1, TIMM50, TIMM8A, TIMMDC1, TINF2, TK2, TKFC, TKT, TLK2, TLR3, TLR4, TM4SF20, TMCO1, TMEM106B, TMEM107, TMEM126B, TMEM127, TMEM138, TMEM165, TMEM216, TMEM231, TMEM237, TMEM240, TMEM43, TMEM63A, TMEM67, TMEM70, TMEM94, TMLHE, TMPRSS6, TMTC3, TMX2, TNF, TNFAIP3, TNFRSF11A, TNFRSF11B, TNFRSF1A, TNFRSF1B, TNFSF11, TNFSF15, TNFSF4, TNIK, TNNT1, TNPO3, TNRC6A, TOE1, TOMM40, TONSL, TOP3A, TOPORS, TOR1A, TP53, TP53RK, TP63, TPI1, TPK1, TPM2, TPM3, TPO, TPP1, TPRKB, TRAF3, TRAF3IP2, TRAF7, TRAIP, TRAK1, TRAPPC11, TRAPPC12, TRAPPC2L, TRAPPC4, TRAPPC6B, TRAPPC9, TRDN, TREM2, TREX1, TRH, TRHR, TRIM2, TRIM32, TRIM37, TRIM71, TRIM8, TRIO, TRIP12, TRIP13, TRIP4, TRIT1, TRMT1, TRMT10A, TRMT5, TRMU, TRNC, TRNE, TRNF, TRNH, TRNI, TRNK, TRNL1, TRNQ, TRNS1, TRNS2, TRNT, TRNT1, TRNV, TRNW, TRPC3, TRPM3, TRPM6, TRPS1, TRPV3, TRPV4, TRRAP, TSC1, TSC2, TSEN15, TSEN2, TSEN34, TSEN54, TSFM, TSHB, TSHR, TSPAN7, TSPYL1, TTBK2, TTC19, TTC37, TTC8, TTI2, TTLL5, TTN, TTPA, TTR, TUB, TUBA1A, TUBA8, TUBB, TUBB2A, TUBB2B, TUBB3, TUBB4A, TUBG1, TUBGCP2, TUBGCP4, TUBGCP6, TULP1, TUSC3, TWIST1, TWIST2, TWNK, TXN2, TXNRD2, TYMP, TYR, TYROBP, UBA1, UBA5, UBAC2, UBAP1, UBE2A, UBE2T, UBE3A, UBE3B, UBQLN2, UBR1, UBTF, UCHL1, UCP2, UFC1, UFD1, UFM1, UFSP2, UGDH, UGP2, UGT1A1, UNC119, UNC13A, UNC80, UNC93B1, UPB1, UPF3B, UQCC2, UQCRQ, UROC1, USF3, USH1C, USH1G, USH2A, USP18, USP27X, USP45, USP7, USP8, USP9X, VAC14, VAMP1, VAMP2, VANGL1, VAPB, VARS, VARS2, VCP, VDR, VEGFC, VHL, VLDLR, VPS11, VPS13A, VPS13B, VPS13C, VPS13D, VPS33A, VPS35, VPS37A, VPS51, VPS53, VRK1, VSX1, VWA3B, WAC, WARS, WARS2, WASF1, WASHC4, WASHC5, WDFY3, WDPCP, WDR11, WDR26, WDR37, WDR4, WDR45, WDR45B, WDR48, WDR62, WDR73, WDR81, WFS1, WHRN, WIPI2, WISP3, WNK1, WNT1, WNT10A, WNT3A, WNT5A, WT1, WWOX, XK, XPA, XPC, XPNPEP3, XPR1, XRCC1, XRCC2, XRCC4, XYLT1, XYLT2, YAP1, YARS2, YEATS2, YIF1B, YME1L1, YWHAE, YWHAG, YY1, YY1AP1, ZBTB11, ZBTB16, ZBTB18, ZBTB20, ZBTB24, ZC3H14, ZC4H2, ZDHHC9, ZEB1, ZEB2, ZFP57, ZFPM2, ZFR, ZFYVE26, ZFYVE27, ZIC1, ZIC2, ZMIZ1, ZMPSTE24, ZMYND11, ZNF142, ZNF148, ZNF365, ZNF408, ZNF41, ZNF423, ZNF462, ZNF469, ZNF513, ZNF592, ZNF711, ZNF81, ZNHIT3, ZSWIM6.*
